# Fasting and non‐fasting plasma levels of monomethyl branched chain fatty acids: Implications for maple syrup urine disease

**DOI:** 10.1002/jmd2.12380

**Published:** 2023-07-14

**Authors:** Trine Tangeraas, Erle Kristensen, Lars Mørkrid, Elisabeth Elind, Yngve Thomas Bliksrud, Lars Eide

**Affiliations:** ^1^ Department of Newborn Screening Oslo University Hospital Oslo Norway; ^2^ Department of Medical Biochemistry Oslo University Hospital Oslo Norway; ^3^ Department of Medical Biochemistry University of Oslo Oslo Norway

**Keywords:** fasting, isoleucine, leucine, monomethyl branched‐chain fatty acids, MSUD, valine

## Abstract

The branched‐chain amino acids (BCAA) leucine, valine, and isoleucine provide precursors for monomethyl branched‐chain fatty acids (BCFA). Established reference ranges for BCFAs are lacking. In maple syrup urine disease (MSUD), a rare inborn error of BCAA metabolism, the endogen production is impaired and MSUD patients are treated with a low protein (low BCAA) diet. The protein restriction may affect the dietary intake of BCFA, depending on the dietary choices made. Patients with MSUD are prescribed a more or less protein‐restricted diet depending on the severity of the disease. The combination of a protein‐restricted diet and subsequent impaired endogenous synthesis may render MSUD patients sensitive to BCFA deficiency, with yet unknown implications. To investigate the possibility of lower circulatory BCFA levels in MSUD that favors dietary BCFA supplementation, we first established fasting‐state reference ranges for selected BCFAs and saturated/unsaturated fatty acids in plasma. Then, the effect of fasting on BCFA levels was evaluated by comparing the distribution in a fasting versus a non‐fasting cohort. To test the hypothesis that BCFA deficiency could contribute to MSUD pathophysiology, we recruited patients with intermittent, intermediate, and classical form of MSUD and analyzed the corresponding BCFA z‐scores. None of the BCFA species had |z‐scores| > 2 relative to the reference range. Our findings do not support the requirement of BCFA supplementation in MSUD patients. The origin of BCFAs is discussed. Impaired capacity to synthesize BCFA do not manifest as reduced plasma levels in MSUD, suggesting that endogenous synthesis is dispensable for plasma levels.

## 1 INTRODUCTION

Monomethyl branched‐chain fatty acids (herein termed BCFAs) are saturated fatty acids with a methyl branch in the penultimate (iso) or antepenultimate (anteiso) position in the carbon chain. The methyl branch modifies the chemical properties of these saturated fatty acids reminiscent of that in unsaturated fatty acids. The BCFAs exist in all organisms and are well known for their important role in temperature adaption in bacteria.^1^ In some bacterial species, the BCFAs constitute more than 20% of the total cellular fatty acids.^2^ In humans, the BCFAs are components of the vernix caseosa providing a dietary source in addition to its structural role as a protective skin‐film for the fetus.^3^ After birth, BCFAs are present in mother's milk, and dairy fat further represent an important dietary source of BCFA in humans (reviewed in Taormina et al.^4^). Degradation of the branched‐chain amino acids (BCAA), leucine, isoleucine, and valine, is necessary for endogenous synthesis of BCFA. The BCAAs are first transaminated by branched‐chain aminotransferase, and the resulting keto acids decarboxylated and conjugated to an acetyl CoA moiety by a large, rate‐limiting metabolon; namely the branched chain keto acid dehydrogenase (BCKD).^5^ The CoA conjugates provide substrates for subsequent BCFA synthesis in the cytosol.^6^

Emerging observations support that the BCFAs have important biological properties for higher organisms including humans. BCFA are associated with anti‐inflammatory properties, exemplified as a protective effect in a rat model of necrotizing enterocolitis^7^ and attenuation of the lipopolysaccharide effect on an in vitro colonocyte model.^8^ Interestingly, while high levels of circulating BCAA are indicative of insulin resistance and Type 2 diabetes,^9^ the inverse is the case for the presence of BCFA in adipose tissue.^10^ Bariatric surgery‐induced weight loss unraveled a positive correlation between insulin sensitivity and BCFA concentration.^11^ Yet another essential biological function of the BCFA in development of *Caenorhabditis elegans* was unraveled by the elegant work of Jia and coworkers.^12^ The underlying mechanism mapped a BCFA‐derived glucosylceramide (d17iso‐GlcCer) to signal apical polarity, which in turn regulates Target of Rapamycin Complex 1 (TORC1) protein.^13^ TORC1 is a central sensor for metabolites and energy, and regulates cell growth and protein synthesis.

In view of the reported biological impacts of BCFA, we speculated that BCFA insufficiency could contribute to the pathology of maple syrup urine disease (MSUD); a rare, inheritable metabolic disorder caused by mutations in the BCKD complex.^14^ Mutations in various subunits of BCKD can cause variants of MSUD disease (classic, intermediate, and intermittent), and manifest with variable biochemical profiles, time of onset of symptoms, metabolic crisis propensity, and sensitivity to protein overload and fasting.^15^ All variants of MSUD are at risk of acute metabolic crisis albeit the risk is highest for the classical form with no residual BCKD activity. Consequences of an untreated metabolic crisis includes brain edema, coma and irreversible brain damage, and even death may ensue if left untreated. Patients with classical MSUD are in particular chronically exposed to fluctuations in the BCAA and are at risk for the development of movement, behavioral, and mood disorders in the long run.^14^ MSUD pathology is linked to toxic effects on neural cells, biochemical pathways, genotoxic effects of keto acids, disturbed BCAA ratios affecting brain signaling pathways, and imbalanced mitochondrial biogenesis,^16^, ^17^, ^18^ while the possible effect of insufficient endogenous BCFA synthesis in MSUD has to our knowledge not been addressed. We previously demonstrated about 90% reduction in intracellular C3 and C5 acylcarnitine levels in primary fibroblasts obtained from patients with intermittent MSUD, thereby supporting an apparent inadequate state of BCFA precursors.^19^ Furthermore, an earlier study showed that submucous BCFA 18‐methyleicosanoic acid was absent in hair follicles from MSUD patients, suggestive of BCFA deficiency.^20^

The aim of the current study was to establish reference ranges for BCFA in a healthy cohort, and to investigate if MSUD patients have reduced endogenous production of plasma BCFA.

## 2 MATERIALS AND METHODS

### 2.1 Ethical approval

Human biological material was collected from two general biobanks approved by the Norwegian South‐East Regional Ethical committee: “General biobank for reference material” (REK 2017/2189) and “General biobank for inborn, rare disorders of metabolism” (REK 2017/2218). The use of biobank material was approved by the Regional ethical committee as part of the following projects: “The establishment of reference ranges for BCFA” (REK 2017/2189), and “Metabolic changes in MSUD” (REK 2017/2190). The general biobank for inborn, rare disorders of metabolism include EDTA‐plasma samples from non‐fasting MSUD patients that were followed‐up at Oslo University Hospital, Oslo, Norway or Haukeland Hospital, Bergen, Norway. The samples were collected and approved for research use upon signed consent by the parents. Included as controls in this biobank are EDTA‐plasma from healthy, non‐smoking, non‐fasting volunteers that permit use of samples for use in research upon signed consent. The general biobank for reference material include EDTA‐plasma obtained from healthy volunteers who had fasted overnight (12 h) upon signed consent for approved use in research.

Immediately after blood sampling, the tube was rapidly mixed and aliquots collected for dried filter spots and the remaining centrifuged (all within 15 min on ice). Supernatant aliquots were stored at −80°C.

### 2.2 Quantification of fatty acids including BCFA in plasma samples

The fatty acids were measured by Vitas AS (www.vitas.no). Plasma samples were thawed in fridge overnight, vortexed, and pipetted into vials. Internal standard (triheptadecanoin) was added and samples methylated with 3 N Methanol—Hydrochloric acid. The resulting fatty acid methyl esters (FAMEs) were extracted with hexane, followed by neutralization. After mixing and centrifugation, the hexane phase was split in two replicates and injected into a Gas Chromatography with Flame Ionization Detection. Analysis was performed on a 7890A gas chromatography instrument with a split/splitless injector, a 7683B automatic liquid sampler, and flame ionization detection (Agilent Technologies, Palo Alto, CA). For the main fatty acids, separation was performed on a SP‐2380 (30 m × 0.25 mm i.d. × 0.25 μm film thickness) column from Supelco. To quantify the BCFA, separations was also performed on a Varian CP7421 (200 m × 0.25 mm i.d.) column from Varian Inc. Identification of BCFAs was done by comparing retention times with standards from Larodan (BR‐2 and BR‐3) and for identification of the main fatty acids by comparing retention times with standard FAME 37 MIX from Supelco.

### 2.3 Statistics

EDTA‐plasma from 123 fasting healthy volunteers were used to establish reference ranges for BCFAs. For each fatty acid, the values (*X*) were ranked in ascending order, assigned percentiles according to Blom's formula: percentile = (rank‐3/8)/(*n* + 1/4) and the associated z‐score (z‐theoretical) in the standard unit normal distribution. As the values exhibited a skewed distribution, they were subjected to a Box–Cox (B–C) transformation with an optimal parameter λ, which rendered a maximal rectilinear correlation (r) between the transformed value (Ξ) and the theoretical z‐score distribution in the 95% central part of the quantile–quantile plot (Q–Q plot). From the intercept A and slope B of the linear regression in the Q–Q plot estimates for the expectation value μ = −A/B, standard deviation s and an experimental z‐score for the value Ξ and (hence also for *X*) could be calculated as Z‐experimental = (Ξ − μ)/s. Outliers with an experimental |z‐score| > 3.37 (Tukey fence factor = 2) were excluded. These parameters (λ, μ, and s) from the transformation of reference cohort set were used to calculate Z‐scores for the non‐fasted cohort (*n* = 20) and for MSUD samples. Reference ranges (2.5 and 97.5 percentiles) are calculated as back‐transformed values of μ − 1.96 s and 1 + 1.96 s.

## 3 RESULTS

To establish reference range for BCFA in plasma, we recruited 123 healthy, non‐smoking volunteers who had fasted overnight (12 h). The age of the reference cohort spanned from 18 to 66 years, with a median age of 38 years. Females were slightly overrepresented (70%). The plasma level of 24 selected fatty acids; saturated, mono‐ and poly‐unsaturated fatty acids and three saturated BCFAs: isoC15, anteisoC15, and anteisoC17 were analyzed by gas chromatography mass spectrometry and the plasma levels for each fatty acid transformed to obtain rectilinear correlation with the theoretical percentile, as described in Section 2. The Supporting Information Figure S1 depicts the Q–Q plots of the individual fatty acids. Table 1 presents reference ranges for identified fatty acids. Spearman correlation analyzes yielded significant correlation between all fatty acids. The odd‐chain saturated fatty acid C15 showed strong correlation with BCFA, in line with previous reports^10^ (Supporting Information Table S1).

**TABLE 1:** Reference range for fatty acids in plasma in 123 fasting volunteers.

	*N*	Median	Lower reference limit 2.5%‐ile	Upper reference limit 97.5%‐ile	λ	Fasting‐induced mean Z‐score shift
BCFA						
iso‐C15	116	3.33	2.99	7.46	0.11	−1.3*
anteiso‐C15	113	2.45	2.67	6.40	−0.12	−0.6*
anteiso‐C17	117	9.29	7.42	12.52	0.16	−0.3*
SFA						
C12	117	10.7	7.51	51.2	−0.65	−1.0*
C14	117	103	51.6	227	−0.39	−0.8*
C15	117	25.8	6.43	29.6	0.23	−0.5*
C16	117	2340	1290	2930	0.59	−0.6*
C18	117	698	456	1029	0.022	−0.8*
C20	117	26.1	20.8	32.3	−0.23	−0.7*
C22	117	68.9	49.6	74.2	−0.083	−0.4*
UFA						
c14_1c9	117	6.38	5.0	17.3	0.34	−1.3*
c161_c9	117	162	84.6	141	0.037	−0.5*
c18_1c9	117	2080	1408	3040	0.21	−0.6*
c18_1c11	117	144	95.6	145	0.39	−0.2
c18_2n6	117	2950	1390	3440	1.0	−0.6*
c18_3n6	117	31.5	34.8	63.5	0.35	−0.5*
c18_3n3	117	71.5	53.8	105	−0.35	−0.9*
c20_1n9	117	18.2	9.15	25.3	−0.87	−0.4*
c20_2n6	117	23.8	12.7	34.7	0.3	−0.6*
c20_3n6	117	128	70.0	173	−0.083	−0.2
c20_4n6	117	563	404	541	−0.14	0.2
c20_5n3	117	123	41.5	142	−0.16	−0.3*
c22_5n3	117	47.3	25.3	64.2	0.12	−0.2
c22_6n3	117	235	135	359	0.29	−0.2

*Note*: Fasting‐induced Z‐score shift was determined from the slope and intercept of transformed values for the reference (in this table) and a corresponding non‐fasting cohort (*N* = 20). Box–Cox λ's shown. Concentrations in nmol/mL plasma.

Abbreviations: SFA: saturated fatty acids, UFA: unsaturated fatty acids.

* Biologically significant effect.

To evaluate the impact of fasting on the levels of fatty acids in circulation, a non‐fasting cohort was included in the study. Plasma from a randomized selection of healthy, non‐smoking volunteers (26–66 years old, median age of 48 years, 70% females, *n* = 20) were collected and analyzed similarly as in the reference cohort, using the same parameters for transformation and calculation of Z‐scores.

The differences in Z‐scores (relative to median of reference value) for the fasted and non‐fasted probability distributions of the fatty acids are provided in Table 1.

**FIGURE 1:**
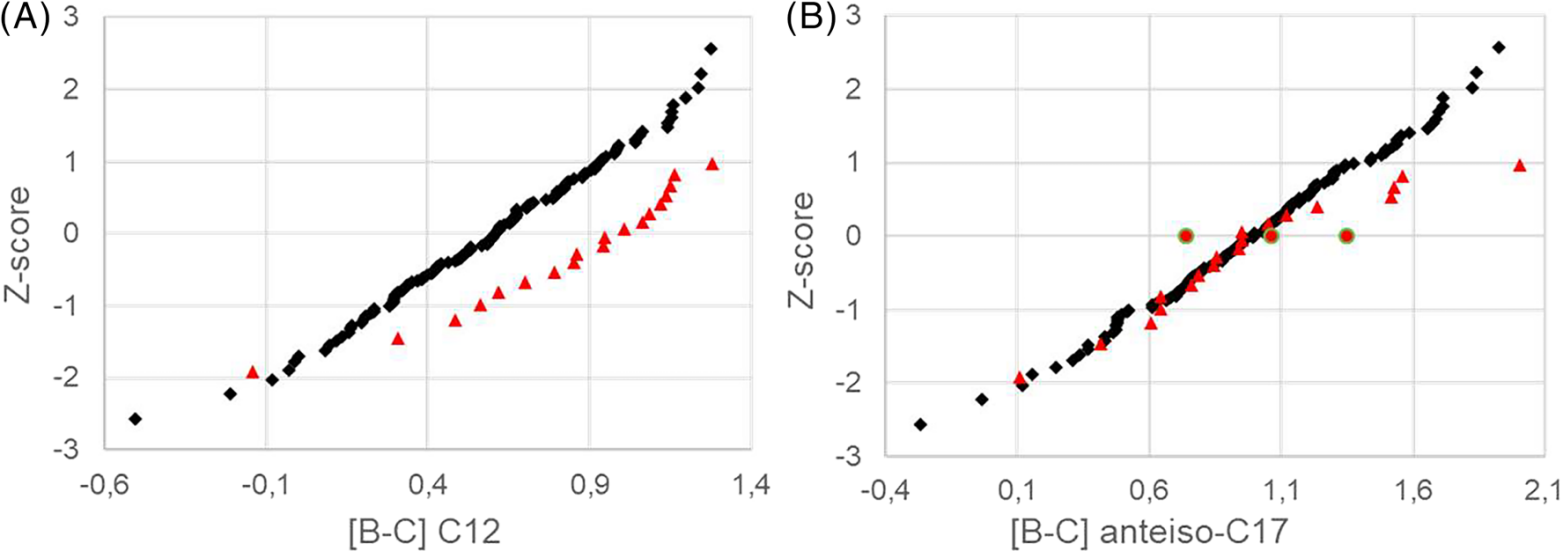
Q–Q plot of transformed C12 (A; left panel) and anteiso‐C17 (B; right panel). Black and red symbols represent the fasting cohort and non‐fasting cohorts, respectively.

As shown in Table 1 most of the identified fatty acids respond to fasting but to a different extent. This is exemplified in Figure 1. The difference in mean Z‐score represent the Cohen's d, which ought to be numerically greater than 0.25 to indicate a biologically significant effect size. The saturated fatty acid, lauric acid (C12) and the BCFA anteiso‐C17 yielded shifts in mean Z‐score of −1.0 and −0.3, respectively (Table 1 and Figure 1).

The BCFA anteiso‐C17 was largely similar in the fasting and non‐fasting reference cohorts. Endogenous synthesis is therefore a possible source for the BCFA anteiso‐C17 and a likely candidate to be significantly affected in disease with impaired BCAA metabolism, such as MSUD.

**FIGURE 2:**
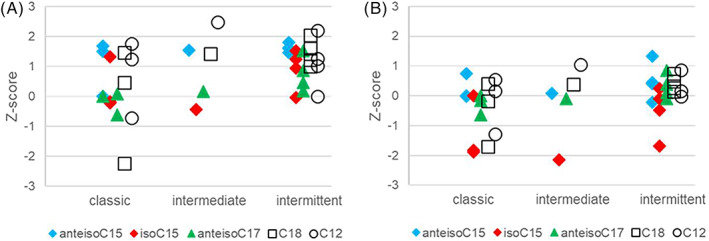
Z‐score of three separate BCFAs in MSUD patients diagnosed as classic (*n* = 3, intermediate (*n* = 1), or intermittent (*n* = 4). The different BCFAs have color codes as indicated. (A): Z‐score calculated relative to fasted reference. (B): Z‐score calculated relative to non‐fasted cohort.

We enrolled MSUD patients of variable disease categories: intermittent (*n* = 4), intermediate (*n* = 1), and classic (*n* = 3) forms of MSUD (Supporting Information Table S2). The fatty acids levels in MSUD patient samples were B–C transformed using the rectilinear coefficients for reference set (Table 1), compared to reference ranges and values from the non‐fasting cohort, as shown in Figure 2A,B, respectively.

None of the selected BCFAs in the MSUD patients were significantly affected relative to the established reference range for the fasting cohort. For some MSUD samples, iso‐C15 levels were borderline low (Figure 2B) independent of disease severity, relative to non‐fasted values.

## 4 DISCUSSION

In this study, we have established reference levels of selected fatty acids and included three BCFA species. Reference ranges for BCFAs are generally lacking, which is an apparent requisite in view of the emerging reports demonstrating distinct roles of BCFAs in biology and medicine. As expected, the levels of BCFA were lower during fasting although the anteiso‐forms were relatively less affected than the iso‐C15 BCFA. Anteiso‐C17 is the most abundant BCFA of the three investigated here. Western lifestyle food (United States) has been analyzed for BCFA content, showing similar levels of anteiso‐C15 and anteiso‐C17,^21^ while, for example, mother's milk has a relative abundance reminiscent of the plasma levels in this study.^22^ Hence, circulating levels of BCFA are probably influenced by other sources than dietary ones, possibly induced by endogenous synthesis.

In the fasted state, levels of circulating branched‐chain C13 fatty acids are 3–4 times lower than lauric acid (C12), while anteiso‐C17 reveals levels several hundred fold lower than palmitate (C16). Spearman's correlation analyses indicated significant correlation between the BCFAs and all straight chain saturated fatty acids (data not shown), and in particular C15 (Supporting Information Table S1). The strong correlation even between fatty acids that are strongly influenced by dietary intake is indicative of a systemic regulation of plasma fatty acid composition.

We did not include a dietary questionnaire, as it was outside the scope of this study. Nogiec and Kasif^23^ reported a 2‐fold increase in isoleucine/leucine levels in postprandial state with a more modest increase in valine level, suggesting that anteiso‐BCFA should respond as much as iso‐BCFA to dietary intake given the endogenous production would depend on available BCAA. Our finding that iso‐C15 differed more between the fasting versus non‐fasting group is in accordance with the findings in rodents, where iso‐fatty acids but not anteiso fatty acids increased postprandially.^6^

The aim of this study beyond establishing BCFA reference values was to deduce whether plasma BCFA insufficiency could be a contributing factor to the pathology of MSUD. We did not find any evidence of reduced plasma BCFA levels in MSUD patients, using the established fasting reference ranges. The majority of z‐scores for BCFA species in MSUD patients were slightly above median (Figure 2A), most likely because blood sampling were obtained at only 3–5 h of fasting as recommended for follow‐up monitoring of the level of branched‐chain aminoacids in MSUD patients, and therefore, probably too short time elapsed to reduce circulating fatty acids. The overall higher levels of C12 and C18 fatty acids in the intermittent MSUD group indicates a shorter fasting period (Figure 2A). Relative to the circulating levels of the non‐fasting cohort (Figure 2B), we discovered close to normal levels of anteiso‐BCFA in MSUD, albeit this was not the case for iso‐C15. We have no reason to assume the lower iso‐C15 level in MSUD is due to limited dietary uptake of BCFA species.^22^ The dietary prescription for all MSUD categories (see Supporting Information) contains lipid sources that may provide dietary intake of fatty acids in particular from diary sources, including iso‐C15 comparable to healthy population. We rather favor the hypothesis that plasma iso‐C15, being most responsive to diet, is provided by a combination of dietary intake and endogenous synthesis. MSUD patients exhibit a low BCAA degradation capacity, even carriers of the mild intermittent form of the disease.^19^ Thus, some MSUD patients demonstrate an apparent iso‐C15 deficiency compared to controls that is caused by inability to support postprandial iso‐C15 endogenous synthesis. We do not believe this finding impacts on health as the iso‐C15 levels do not drop below fasted‐state levels. However, we cannot rule out regulatory roles of iso‐C15 and similar compounds have impact in the non‐fasting situation in MSUD. Since BCFA and BCAA are inversely correlated, it can be speculated that BCFA in the postprandial phase could stimulate BCAA uptake, similar as insulin, which has been used for MSUD patients.^24^

Cerebrospinal fluid levels of BCFA would be a preferred material to investigate the impact of BCFA on MSUD pathology. Plasma BCFA is at best only a surrogate marker for brain levels and cannot be used to conclude that BCFA is dispensable for MSUD pathology. Although it is expected that the barrier between the peripheral blood and brain tissue (blood–brain barrier) and the blood–cerebrospinal fluid barrier provides fatty acid exchange, it is to our knowledge not described how these BCFA are distributed over these barriers. The normal plasma levels of BCFA nevertheless demonstrates that dietary therapy is unnecessary therapy for MSUD. Another apparent limitation is that the MSUD patients do not age‐correlate with the control group. The controls, however span almost 40 years, and as many fatty acids exhibited a positive correlation with age, including anteiso‐C17 (R = 0.22, *p* < 0.01) but not iso‐C15 or anteiso‐C15, we do not believe this inconsistency would weaken our conclusion.

In summary, we have established reference ranges for three BCFA species: iso‐C15, anteiso‐C15, and anteiso‐C17 representing fatty acids of isoleucine and leucine/valine, respectively. We observed lower levels than expected in non‐fasting state, suggesting that endogenous synthesis from dietary amino acids contributes to the food‐associated increase of some BCFA species. Our findings is partly contradictory to the reports of lower BCFA in hair follicles of MSUD patients and that adipocytes in mammals are capable of producing large amounts of BCFA. Possibly, the explanation is that local tissue levels of BCFA differ from circulatory levels. We did not address the role of microbiota as providers of BCFA in this study, but microbiota's contribution to diet‐independent levels of BCFA awards investigation^22^ and could contribute to the diet‐independent levels of BCFA.

## CONFLICT OF INTEREST STATEMENT

The authors declare no conflicts of interest.

## Supporting information


**Data S1:** Supporting Information.

## Data Availability

De‐identified data are available from the authors upon request.
