# Supplementary material for: Fasting and non‐fasting plasma levels of monomethyl branched chain fatty acids: Implications for maple syrup urine disease
Source: JIMD Rep. 2023 Jul 14;64(5):360–6. doi: 10.1002/jmd2.12380 (PMC10494493; doi:10.1002/jmd2.12380)
Supplement: Supplementary file 1 — Data S1: Supporting Information. [file JMD2-64-360-s001.docx]

Supplementary information

Dietary regimes for MSUD patients in the study.

Dietetic Management of MSUD in general includes variable restriction of natural protein dependent on the disease severity. Classic MSUD dietetic management includes a strict low protein diet supplemented with leucine, valine and isoleucine-free amino acids.Protein restriction is adjusted based on plasma BCAA values. Valine and isoleucine is supplemented if necessary and adjusted according to plasma levels, as described (Strauss et al 2020). The BCAA free amino acid supplements used to secure sufficient amino acid intake by the patients in this study was MSUD gel (Vitaflo, Nestlè, Switzerland) and MSUD Anamix Infant (Nutricia, Danone, Brussel). The MSUD Anamix Infant is used as a BCAA free breast milk substitute as well as a BCAA free amino acid supplement for older children. Both contain fat from vegetable oils and single cell oils (securing the intake of DHA) as well. There is no Dairy Fat added to the BCAA free amino acids supplements. The ingredients list is shown hereunder.

Moderate protein restriction includes less strict natural protein restriction with or without branched-chain free amino acids supplementation. A low protein diet includes preference of cow milk with higher fat/protein ratio such as cream and sour cream as well as low protein cream containing products like ProZero (Vitaflo, Nestlè) and LoProfin Drink LQ (Nutricia, Danone)(Shaw 2020) .

MSUD Gel contains sucrose, starch, L-lysine L- aspartate,L-arginine L-aspartate, L-leucine, L-glutamine, modified starch, L-alanine, L-serine, L-histidine, L-tyrosine, L-phenylalanine, tri calcium phosphate, L-proline, glycine, L-cystine, calcium glycerophosphate, trisodium citrate, magnesium hydrogen phosphate, L-tryptophan, potassium chloride, tripotassium citrate, choline bitartrate, maltodextrin, L-isoleucine, L-ascorbic acid, taurine , sodium chloride, zinc gluconate, L-carnitine, L-tartrate, ferrous sulphate, D/L-alpha tocopheryl acetate, nicotinamide, manganese gluconate, chromium chloride, sodium selenite, retinyl acetate, vitamin D3 (cholecalciferol), copper gluconate, D-calcium pantothenate, biotin, folic acid, vitamin B12 (cyanocobalamin), potassium iodide, pyridoxine hydrochloride, thiamin hydrochloride, sodium molybdate, riboflavin, vitamin k (phylloquinone).

MSUD Anamix Infant contains corn syrup solids, refined vegetable oils (high oleic sunflower, soy, non-hydrogenated coconut, canola, sunflower), galacto-oligosaccharides (Milk), L-lysine L-glutamate, L-proline, L-arginine, glycine, L-aspartic acid, tricalcium phosphate, L-threonine, citric acid esters of mono- and diglycerides, L-phenylalanine, L-serine, L-histidine, L-alanine, potassium chloride, fructo-oligosaccharides, tripotassium citrate, L-cystine, L-tryptophan, sodium phosphate dibasic, magnesium acetate, L-methionine, M. Alpina Oil*, choline bitartrate, C. Cohnii Oil**, L-glutamine, sodium chloride, inositol, L-ascorbic acid, L-glutamic acid, taurine, ferrous sulfate, zinc sulfate, L-carnitine, DL-alpha tocopheryl acetate, calcium D-pantothenate, niacinamide, ascorbyl palmitate, manganese sulfate, cupric sulfate, thiamine hydrochloride, pyridoxine hydrochloride, riboflavin, vitamin A acetate, DL-alpha tocopherol, mixed tocopherols, potassium iodide, chromium chloride, folic acid, phylloquinone, sodium selenite, sodium molybdate, D-biotin, vitamin D3, cyanocobalamin.

Loprofin Drink LQ contains cream, whey and lactose (from cow`s milk powder), emulsifier (E471), mono- and diglycerides of fatty acid: 2.1 g fatty acid per 100 mL.

Supplementary Table 1

Spearman correlation analyses

| BCFA |  | anteiso-C15 | anteiso-C17 | C15 |
| --- | --- | --- | --- | --- |
| iso-C15 | r  p-value  n | 0,57  1,9E-11  117 | 0,52  9,5E-10  120 | 0,517  1,4E-9  120 |
| anteiso-C15 | r  p-value  n |  | 0,82  1,8E-29  117 | 0,784  1,5E-25  117 |
| anteiso-C17 | r  p-value  n |  |  | 0,838  4,7E-33  121 |

R: correlation coefficient (p-value: 2-tailed Spearman analyses)

Supplementary Table 2

MSUD patient samples included

| **MSUD type** | **Reference sequence** | **Allele 1** | **Allele 2** | **Gender** | **Age at sampling** | **Dietetic management** |
| --- | --- | --- | --- | --- | --- | --- |
| Classic | NM_000709.3  (BCKDHA) | c.375+ 648_484 + 520del p.Gly126Valfs*3 | c.375+ 648_484 + 520del p.Gly126Valfs*3 | M | 5 | **Protein restriction** |
| Classic | Unknown; diagnosis based on cellular 14C-Leucine degradation | n.a. | n.a. | M | **2** | **Protein restriction** |
| Classic | NM 001918.2 (DBT) | c1111 dup  p.(Ala371GlyfsTer3) | c1111 dup  p.(Ala371GlyfsTer3) | F | 10 | **Protein restriction** |
| intermittent | NM_001918.3  (DBT) | c.901C>T (p.Arg301Cys) | c.1291 C>T (p.Arg431*) | F | 5 | **Moderate protein restriction** |
| intermittent | NM_001918.3  (DBT) | c.901C>T (p.Arg301Cys) | c.1291 C>T (p.Arg431*) | F | 4 | **Moderate protein restriction** |
| intermittent | NM_001918.3 (DBT) | c.901C>T (p.Arg301Cys) | c.*358A>C | M | 17 | **Moderate protein restriction** |
| intermittent | NM_001918.5  (DBT) | c.75_76del  (p.Cys26fs) | c.827T>G (p.Phe276Cys). | F | 9 | **Moderate protein restriction** |
| Intermediate | NM_001918.5  (DBT) | c.1126C>T (p.Arg376Cys | c.1126C>T (p.Arg376Cys | F | <1 | **Moderate protein restriction** |

**
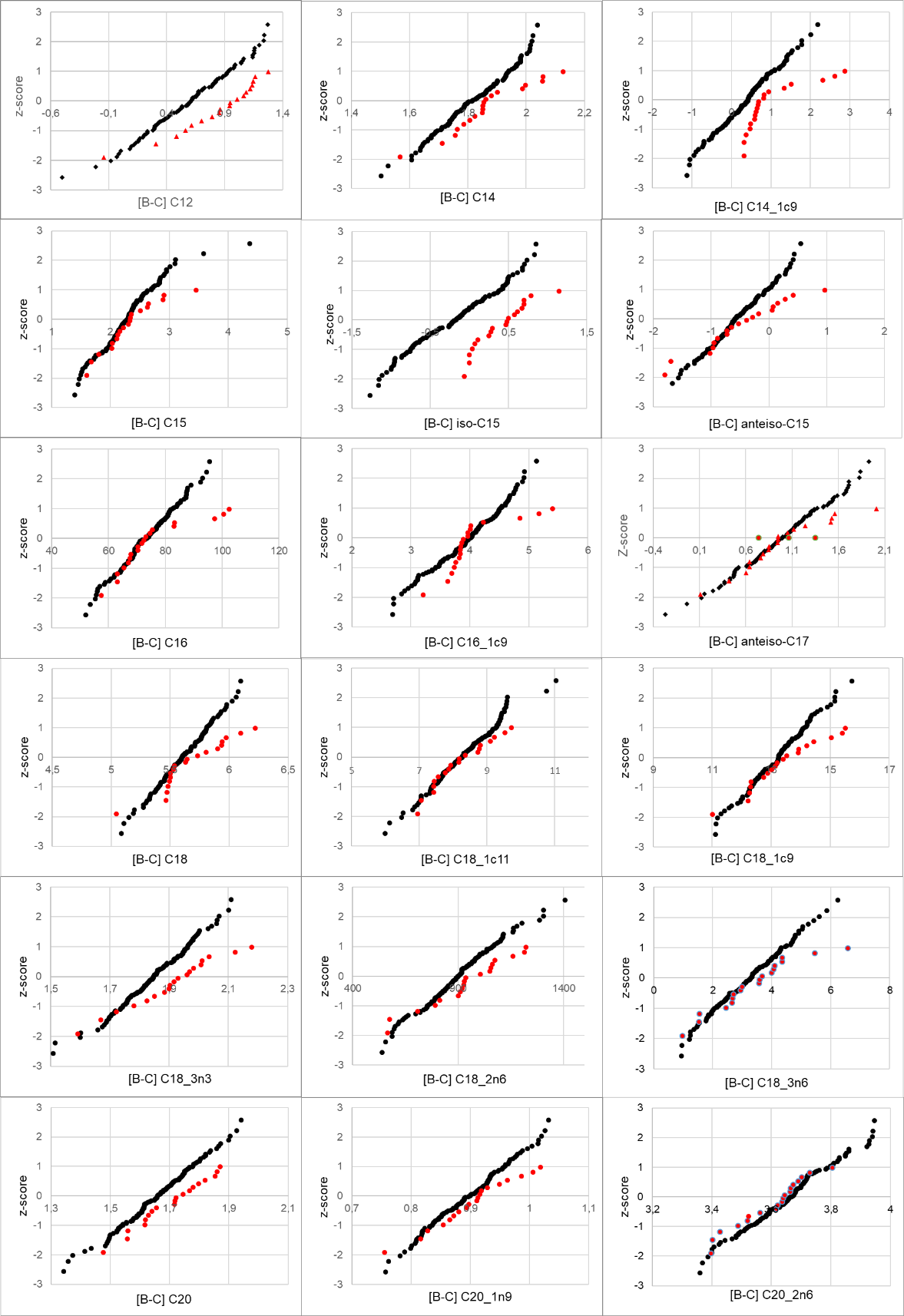
**Supplementary Figure 1

Calculated z-scores of the identified fatty acids concentration after Box-Cox transformation. The Q-Q plots depict transformed values from fasted (black) and non-fasting (red) cohorts. The medians, peripheral percentiles and Box-Cox λ’s are provided in Table 1.
